# Effect of prolonging radiation delivery time on retention of gammaH2AX

**DOI:** 10.1186/1748-717X-3-18

**Published:** 2008-06-27

**Authors:** Vitali Moiseenko, Judit P Banáth, Cheryl Duzenli, Peggy L Olive

**Affiliations:** 1 Medical Physics Department, British Columbia Cancer Agency, Vancouver, Canada; 2 Medical Biophysics Department, British Columbia Cancer Research Centre, Vancouver, Canada

## Abstract

**Background and purpose:**

Compared to conventional external beam radiotherapy, IMRT requires significantly more time to deliver the dose. Prolonging dose delivery potentially increases DNA repair which would reduce the biological effect. We questioned whether retention of γH2AX, a measure of lack of repair of DNA damage, would decrease when dose delivery was protracted.

**Materials and methods:**

Exponentially growing SiHa cervical carinoma cells were irradiated with 6 MV photons in a water tank using a VarianEX linear accelerator. Cells held at 37°C received 2 Gy in 0.5 min and 4 Gy in 1 min. To evaluate effect of dose delivery prolongation, 2 and 4 Gy were delivered in 30 and 60 min. After 24 h recovery, cells were analyzed for clonogenic survival and for residual γH2AX as measured using flow cytometry.

**Results:**

Increasing the dose delivery time from 0.5 or 1 min to 30 or 60 min produced a signficant increase in cell survival from 0.45 to 0.48 after 2 Gy, and from 0.17 to 0.20 after 4 Gy. Expression of residual γH2AX decreased from 1.27 to 1.22 relative to background after 2 Gy and 1.46 to 1.39 relative to background after 4 Gy, but differences were not statistically significant. The relative differences in the slopes of residual γH2AX versus dose for acute versus prolonged irradiation bordered on significant (p = 0.055), and the magnitude of the change was consistent with the observed increase in surviving fraction.

**Conclusion:**

These results support the concept that DNA repair underlies the increase in survival observed when dose delivery is prolonged. They also help to establish the limits of sensitivity of residual γH2AX, as measured using flow cytometry, for detecting differences in response to irradiation.

## Background

Intensity modulated radiation therapy (IMRT) is being adopted in radiotherapy centers world-wide with the goal of improving beam conformation to the tumor while minimizing damage to surrounding normal tissues. However, IMRT treatments require longer dose delivery times leading to the concern that less tumor cell kill will occur as a result of repair during treatment [1,2]. Experiments from several groups have demonstrated small but often significant increases in cell survival when dose delivery is protracted over 10–30 min [3-6].

The increase in cell survival by protraction of dose has been associated with the capacity of cells to repair DNA damage; one of the earliest papers reported a reduction in chromsome aberration frequency when the X-ray dose was delivered in 1 min versus 16 min [7]. Therefore, IMRT should be less effective because fewer lethal DNA lesions are produced for the same total dose [8]. While experimental evidence using survival is convincing, a direct measure of DNA repair provides insights into the effect of dose delivery prolongation in IMRT and conditions under which this prolongation would be detrimental for radiation therapy outcome.

DNA double-strand breaks are generally accepted to be the most important potentially lethal lesions produced by ionizing radiation. A method for the sensitive detection of individual double-strand breaks is based on measurement of a specific phosphorylation on a nucleosomal histone, H2AX, that occurs rapidly at the site of each double-strand break [9]. Antibody labeling of the phosphorylated form of H2AX (called γH2AX) together with flow cytometry provides a rapid and objective method of quantifying this molecule after irradiation [10]. In contrast to other DNA damage assays (e.g., pulsed-field gel electrophoresis or comet assay), γH2AX can be used to detect double-strand breaks at therapeutic doses. The fraction of tumor cells that retain γH2AX foci 24 h after X-irradiation has been correlated with the fraction of cells that survive to form a colony [11,12]. The question we wished to address is whether flow cytometry analysis of residual γH2AX would be sufficiently sensitive and robust to detect the relatively small increase in surviving fraction after protracted radiation exposures. We chose to examine SiHa cervical carcinoma cells since this cell line showed a significant increase in surviving fraction using an IMRT protocol [5]. Preliminary experiments using continuous low dose rate versus acute exposure to X-rays [13] provided the rationale for developing a protocol to simulate an IMRT dose delivery rate using a linear accelerator. The overall dose delivery time was longer than is typical for IMRT, but the major goal was to employ a simple and well defined dose delivery method to test the ability of γH2AX to serve as a surrogate of cell killing.

## Methods

### Cell source and maintenance

SiHa human cervical cancer cells were obtained from American Type Culture Collection and maintained in minimal essential medium (MEM) containing 10% fetal bovine serum (FBS). Exponentially growing cells were seeded overnight in 90 mm dishes and trypsinized just before each experiment using 0.1% trypsin in citrate saline buffer. Single cells were resuspended in 5 ml MEM + 10% FBS at a density of 2 × 10^5 ^cells/ml in Falcon polycarbonate tubes. Six tubes were prepared for each exposure condition and transferred to a water bath at 37°C.

### Irradiation

Tubes were submersed and irradiated in a water tank at 37°C as an array of six with 6 MV photons using Varian EX linear accelerator. Irradiation conditions were: source-axis distance 100 cm, depth 5 cm, field size 20 × 20 cm^2^. Tubes were placed within a 10 cm region, 10 cm from the tube midline to the bottom of the tank for backscatter. The photon beam was calibrated according to the TG51 protocol; output variations through the time period spanning measurements were within 0.3% according to monthly quality assurance. Irradiations were performed at a rate of 400 MU/min, with acute doses of 2 and 4 Gy delivered in 0.5 and 1 min. For protracted delivery, 16 equal segments were delivered with 2 and 4 min intervals between the segments for the 30 and 60 min deliveries, respectively. Splitting the total dose into multiple small doses as a surrogate for IMRT was previously used by Ogino et al. [14]. Although only one fractionation schedule was examined here, this was considered adequate to determine whether γH2AX could be a useful surrogate for cell clonogenic response.

### Clonogenic survival assay

After exposure, cells were plated in 60 mm tissue culture dishes and allowed to recover for 24 h while attached to plates. This delay in plating was necessary so that the response of the same cell population could be compared for retention of γH2AX and survival. After recovery, cells were trypsinized and counted with a Coulter Counter. A portion of the cells was plated at a density of 500–2500 cells in duplicate using 90 mm dishes. Plates were stained and colonies were counted two weeks later. Counts from the two plates were averaged, and surviving fraction was calculated as the ratio of the plating efficiency of the treated cells divided by the plating efficiency of the control cells (0.68 ± 0.02). Experiments were repeated 2–4 times giving results for 12–24 irradiated tubes. From each experiment, the remaining cells were fixed in 70% ethanol for measurement of residual γH2AX.

### Measurement of γH2AX by flow cytometry

Ethanol-fixed samples were rehydrated in Tris-buffered saline and incubated for 2 hours with anti-γH2AX mouse monoclonal antibody (Upstate or Abcam, 1:500 dilution). Samples were washed by centrifugation and resuspended for one hour in secondary Alexa-488 conjugated goat anti-mouse IgG (Molecular Probes, 1:200). After a second rinse, cells were resuspended in 1 μg/ml ml 4',6-diamidino-2-phenylindole dihydrochloride hydrate (DAPI; Sigma) to stain DNA. Analysis was conducted using a dual-laser Coulter Elite cell sorter [15]. Samples were gated based on forward and peripheral light scatter, and 10,000 cells were analyzed. To correct for differences in DNA content, FITC fluorescence intensity was divided by DAPI fluorescence intensity for each cell. Relative γH2AX fluorescence was then determined by dividing the mean fluorescence of the irradiated samples by the mean fluorescence of the control samples, averaged for the 3–6 control samples per individual experiment. Two to 4 experiments were conducted per dose and data were pooled.

### Statistical analysis

ANOVA was used to determine the significance of the difference between variation for inter-experiment versus intra-experiment measurements. Analysis of co-variance was used to examine the significance of the difference between the slopes of the γH2AX dose response curves for acute versus protracted irradiations.

## Results and discussion

**Figure 1:**
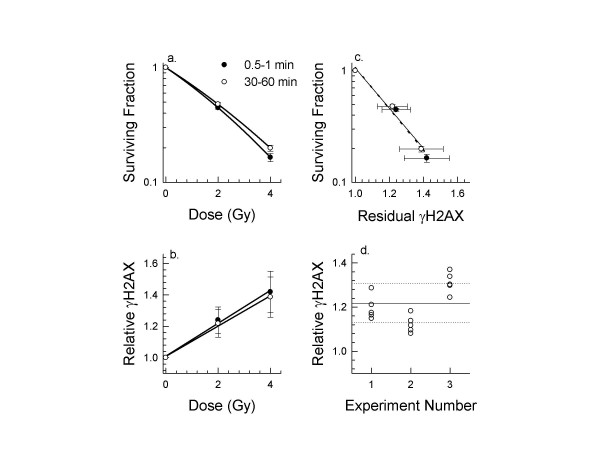
**Comparison between surviving fraction and relative retention of γH2AX for SiHa cells exposed to 2 or 4 Gy over 0.5 or 1 min or protracted over 30 or 60 min**. Panel a: Clonogenic surviving fraction (mean ± SD). Panel b: Residual γH2AX relative to unirradiated cells (mean ± SD). Panel c: Comparison between results in panel a and b. Linear best fits are shown for the acute and protracted exposures. Panel d: Relative γH2AX calculated for 3 independent experiments of 6 samples when SiHa cells were exposed to 2 Gy using the protracted protocol. Mean ± SD for the pooled samples is indicated by the lines. The F value is 18.95 indicating significant inter-experimental differences.

Clonogenic surviving fraction was measured for SiHa cells exposed to 2 or 4 Gy given either within 1 min or using an protocol that protracted dose delivery over 30 or 60 min. No difference in clonogenic survival was observed between the responses of cells exposed using the 30 or 60 min protocols so those results were pooled. The lack of a difference between 30 and 60 min is not unexpected because the dose per fraction is small enough to ensure that the alpha term dominates the response. Prolonging the dose delivery resulted in a significant increase in clongenic surviving fraction for SiHa cells (Fig. 1a; Table 1). Although differences in effects of dose protraction are known to be cell line dependent [5], the extent of recovery seen for SiHa cells is within the range of values reported for 7 cell lines [3-6]. Pooling results from these 4 studies, the average increase in surviving fraction for a 2 Gy exposure protracted over 10–30 min was 17 ± 12%. Our results indicate a relative increase of 7.2% when 2 Gy was protracted over 30–60 min. A larger increase might have been observed had cells been plated immediately. The dose deficit over a course of 30 fractions is calculated to be 5.6 Gy, or 3 fractions, even for this small increase.

**Table 1:** Clonogenic survival and γH2AX retention for SiHa cells after acute or protracted exposures

**Dose**	**Integral Exposure Time (min)**	**Surviving Fraction^a ^(number of samples)**	**Residual γH2AX^b ^(number of samples)**
2 Gy acute	0.5	0.446 ± 0.013 (12)	1.265 ± 0.089 (12)
2 Gy protracted	30 and 60	0.478 ± 0.018 (18)*	1.218 ± 0.087 (18)
4 Gy acute	1	0.164 ± 0.014 (18)	1.461 ± 0.142 (12)
4 Gy protracted	30 and 60	0.199 ± 0.012 (24)*	1.387 ± 0.128 (18)

^a ^Mean and standard deviation for clonogenic surviving fraction expressed relative to the unirradiated control cells. Cells were plated for survival 24 h after irradiation.

* Result significantly different relative to the response of cells exposed in ≤ 1 min.

^b ^Mean and standard deviation for γH2AX expression measured 24 h after irradiation and expressed relative to the unirradiated cells.

When cells from the same exposed population were fixed, stained for γH2AX, and analyzed by flow cytometry, more residual γH2AX was observed after acute than protracted exposure (Table 1). Differences between the averages for individual doses were not significant although analysis of the slopes of the two dose response curves in Fig. 1b yeilded a borderline p-value of 0.055. The slope for the acute exposure curve was 8% higher than the slope for protracted delivery, consistent with the observed survival difference. When survival and residual γH2AX were directly compared, the slope was the same for cells exposed acutely to 2 or 4 Gy versus cells given the protracted exposure (Fig. 1c). This result supports the hypothesis that prolonging dose delivery results in reduction of lethal DNA damage and this underlies the increase in cell survival.

Sources of variability in our measurement of γH2AX were largely the result of inter-experimental variability (Fig. 1d). Some of this variability arose from differences in antibody source although normalizing results for each set of experiments did not result in statistically significant differences between the groups. Variation was also associated with daily differences in flow cytometer set-up and alignment. In previous experiments that examined residual γH2AX in human lymphocytes 24 h after 2.7 Gy, ethanol-fixed samples were stored so that samples from 40 patients could be stained and analyzed on the same day [10]. Although this is recommended practice when small differences in response are expected, it is not always practical.

## Conclusion

Results presented here help to define the lower limit of sensitivity of γH2AX for detecting differences in response to radiation using flow cytometry. Differences of 5% or more in surviving fraction for the acute and protracted protocols are likely to be required to detect a significant difference in retention of γH2AX. Under the less than ideal conditions for this study, the extent of reduction in residual γH2AX was consistent with the measured increase in survival. These results support models that explain dose protraction based on DNA repair that occurs during treatment, and they contribute to the increasing experimental evidence indicating that dose correction is advisable when fraction delivery time is increased. Future experiments that examine different fractionation schedules, response of DNA repair deficient cell lines, and behavior of cells *in vivo *should be informative.

## Competing interests

The authors declare that they have no competing interests.

## Authors' contributions

VM conceived the idea and VM, CD and PO designed the experiments, VM and CD performed the irradiations, JB prepared the cells and performed the measurements of γH2AX, PO analyzed the data and drafted the manuscript with the help of VM and JB. All authors read and approved the final manuscript.
